# Space, time, and language

**DOI:** 10.1007/s10339-018-0878-1

**Published:** 2018-08-20

**Authors:** Michael C. Corballis

**Affiliations:** School of Psychology, University of Auckland, Auckland, New Zealand

## Abstract

Cognition is heavily grounded in space. As animals that move in space, we travel both physically and mentally in space and time, reliving past events, imagining future ones, and even constructing imaginary scenarios that play out in stories. Mental exploration of space is extraordinarily flexible, allowing us to zoom, adopt different vantage points, mentally rotate, and attach objects and sense impressions to create events, whether remembered, planned, or simply invented. The properties of spatiotemporal cognition depend on a hippocampal–entorhinal circuit of place cells, grid cells and border cells, with combinations of grid-cell modules generating a vast number of potential spatial remappings. The generativity of language, often considered one of its defining properties, may therefore derive not from the nature of language itself, but rather from the generativity of spatiotemporal scenarios, with language having evolved as a means of sharing them. Much our understanding of the hippocampal–entorhinal circuit is derived from neurophysiological recording in the rat brain, implying that the spatiotemporal cognition underpinning language has a long evolutionary history.

As animals that move, we are supremely adapted to knowing where we are in space, as well as knowing where we have been and where we might go next. These capacities depend not only on immediate sensory input, but also on imagination; we can travel mentally in space and time, replaying past episodes in our lives and imagining future ones, bringing to mind events that have not actually occurred. This is important not only for recording which places and events are dangerous or fruitful, but also for planning futures, perhaps mentally creating different possible scenarios to establish choices for future consideration. Mental travel can also free us from reality, allowing us to create stories and imaginary adventures. In this respect, it is an aspect of play (Boyd, 2009).

Many have argued that mental time travel is unique to humans, with other animals being effectively stuck in the present. Tulving (1972) has long argued that episodic memory, the capacity to consciously replay the past, is restricted to our own species, and Suddendorf and I extended this proposition to mental travels both forward and backward in time (Suddendorf & Corballis, 2007). This was anticipated by Donald (1991) who wrote that “The lives of apes are lived entirely in the present” (p. 149), and much earlier Kohler (1917/1927), based on his studies of problem solving in chimpanzees, wrote that “the time in which chimpanzees live is limited in past and future” (p. 272). More recently, the idea that mental time travel is uniquely human has been strongly challenged by ethologists and animal psychologists, who have offered behavioral evidence for episodic memory and/or future thinking in nonhuman animals; these include birds (e.g., Clayton et al. 2003), chimpanzees (e.g., Janmaat et al. 2014), rats (Wilson et al. 2013), and even cuttlefish (Jozet-Alves et al. 2013).

Behavioral evidence, though, is often ambiguous, and can sometimes be explained in terms of processes other than mental time travel, such as trial-and-error learning or simple association. Part of the difficulty is that behavioral observations may not reveal what Tulving (1985) calls the *autonoetic* aspect of mental time travel, which allows one to consciously place oneself in different places and times and examine one’s own experience. Autonoetic memories contrast with *noetic* memories, which may be consciously accessed but lack the sense of self-reference, and with *anoetic* memories made up of unconscious habits. In animals without language, it is difficult to distinguish the autonoetic from the noetic; for example, an animal may know where food has been cached but have no memory of the act of caching it.

Better information as to the subjective aspect of mental time travel in nonhuman animals may come from actual recordings of brain activity, and correspondences with human brain activity. Recent understanding of the functions of the hippocampus, in particular, suggests a growing rapprochement between human and animal research, and a sense of the evolutionary continuity of mental time travel. It was this information that caused me to change my view and accept the likelihood that mental travels in space and time may long precede human evolution (Corballis 2013; but see Suddendorf 2013, for dissenting opinion).

## Role of the hippocampus

In humans, the hippocampus plays a critical role in both episodic memory and episodic future thinking. Patients with destruction of the hippocampus are unable to recall past events or imagine future ones (Corkin 2013; Wearing 2005). Brain imaging also shows the hippocampus to be active when people are asked to recall previous episodes, or imagine future ones (Addis et al. 2011), or even construct fictitious ones (Hassabis et al. 2007). This is not to say that representations are stored in the hippocampus; rather, the hippocampus may be responsible for scene construction (Maguire et al. 2016), drawing on information stored in many regions of the brain but allowing for vivid mental construction and reconstruction of events. Without that construction, we have no autonoetic sense of past or imagined events, but may well have access to general knowledge (noetic information).

For many years, it was thought that the hippocampus must play a very different role in nonhuman animals. In the rat hippocampus, for example, recordings from single cells, known as “place cells,” correspond to the animal’s location; as the animal moves about, different place cells become active. This led to the notion of the hippocampus as a “cognitive map,” or a kind of internal GPS system (O’Keefe and Nadel 1978). It now seems clear, though, that place cells themselves play a role in episodic memory, and probably in mental time travel generally.

Experiments have shown that hippocampal activity may persist in short-wave ripples (SWRs) after the animal has been removed from a spatial environment, such as a maze, and these ripples map out trajectories in the environment. These trajectories are sometimes “replays” of trajectories previously taken, sometimes the reverse of those trajectories, and sometimes trajectories the animal did not take, some of which may be anticipations of future trajectories. This evidence offers some reconciliation with the evidence from humans. Reviewing the evidence, Moser et al. (2015) write that “the replay phenomenon may support ‘mental time travel’ … through the spatial map, both forward and backward in time (p. 6).” Mental time travel, then, far from being unique to humans, may be present even in the rat, and may go far back in the evolution of moving animals.

Recordings from the rat hippocampus also reveal what have been termed “time cells,” which respond in coordinated fashion to code the relative times in which events occurred. The pattern itself changes over time as the temporal context changes (Eichenbaum 2017). This can be observed experientially in our own memories of when things happened, gradually losing immediacy and detail, both spatial and temporal. Moser et al. (2015) also summarize evidence that place cells in the rat hippocampus respond not only to specific locations, but also to features of environments they have explored, such as odors, touch sensations, and the timing of events. This suggests that the hippocampus acts not only as a cognitive map, but also as a template for mental time travel that includes episodic nonspatial elements. Similar properties apply to hippocampal action in humans. In one study, human patients about to undergo surgery had electrodes implanted in cells in the medial temporal lobe, in an attempt to locate the source of epileptic seizures. They were given the task of navigating a virtual town on a computer screen, and delivering items to one of the stores in town. They were then asked to recall only the items, and not the location to which they were delivered. The act of recall, though, activated place cells corresponding to that location, effectively mirroring the replay of place-cell activity in the rat brain (Miller et al. 2013).

Experimental limitations mean that the evidence from the rat covers short durations of not more than a few hours, whereas human episodic memories can go back years, if not decades, but the commonalities between human and nonhuman hippocampal function suggest that episodic mental travel may long predate human evolution.

## Modulation of hippocampal function

Hippocampal firing is modulated by cells in the adjacent entorhinal cortex. These cells respond to fields laid out in a grid pattern, and different grid-cell modules are dedicated to different geometric aspects of the environment and its relation to the animal’s location. These various properties allow rapid adjustment of the internal map to accommodate changes in viewing angle, relation to contours, and zooming. One set of grid cells codes for head direction, essentially calibrating which way to the animal is facing. These appear to play a role in spatial memory as well as in online spatial awareness, at least in humans. Brain imaging shows that as people navigate in a virtual environment, hippocampal activity peaks in 60-degree steps, concordant with the hexagonal layout grid-cell receptive fields (Doeller et al. 2010). The adjustment of orientation in imagined space is illustrated in a classic experiment in which patients with left hemineglect, a condition in which damage to the right hemisphere results in failure to attend to objects or events on the left side of space, were asked to imagine themselves in the Piazza del Duomo in Milan. They were first asked to imagine themselves at one end of the square facing the cathedral and to list the landmarks they could identify. They systematically neglected those on the left. When then asked to imagine themselves at the cathedral end, facing the other way, they systematically neglected those they had previously identified, now on the left, and identified those they had previously neglected (Bisiach and Luzzatti 1978). This is a striking demonstration not only that spatial orientation is highly flexible, but also that spatial deficits are manifest in imagined as well as in perceived space.

Grid cells also modulate spatial scale, such that different levels of spatial resolution are represented along the axis of the hippocampus, ranging from a more detailed, close view at one end toward a broader, more distant view at the other. In the rat, the dorsal end operates within a region of about 1 m in width, while at the rostral end the width is about 10 m (Kjelstrup et al. 2008). This arrangement allows for zooming, which is ubiquitous in human spatial understanding: we can locate ourselves in the immediate environment, such as an office, or zoom out to understand where we are in a building, a city, a country, or even in a world map. We can also zoom in the imagined past or future, perhaps imagining being in a particular café in Paris, then zooming out to locate the café in that city, and the city in the country. Variations in scale may apply also to our understanding of the structure of events in the world as well as simply to spatial awareness. In one study, people shown sequences of four videos of different events, along with narratives describing the events. At one level, narratives were linked to each video, encouraging attention to individual details. At the next level, narratives linked a pair of videos, and at the final level a narrative linked all four videos. As the people processed these narratives, activation in the hippocampus progressed from the posterior to the anterior end as the scales of the narrative shifted from small and detailed to larger and more global (Collin et al. 2015). This shift probably occurs as you read a novel, with specific information registering as you read each page, but more global understanding as you proceed and later remember the novel.

The entorhinal grid system appears to operate in modular fashion, in which the combinations of just a few modules can generate a vast number of patterns of activity in the hippocampus, since grid modules can assume different levels. Moser and her colleagues write “The mechanism would be similar to that of a combination lock in which 10,000 combinations may be generated with only four modules of 10 possible values, or that of an alphabet in which all words of a language can be generated by combining only 30 letters or less (p. 11).” This combinatorial system may apply across time as well as space, providing for the “hierarchical organization of space, time and [episodic] memory” (Collin et al. 2017)—and no doubt of imagined future episodes as well. In short, the mechanism appears to be both generative and hierarchical. As such, it may well underlie the organization of language itself.

## Language

One of the essential properties of language is generativity, the capacity through hierarchical representations to produce a potentially infinite number of sentences. According to Chomsky, this capacity is unique to humans, emerging within the past 100,000 years or so. Its primary operation is “unbounded Merge” (e.g., Berwick and Chomsky 2015; Chomsky 2010), whereby symbolic structures can be progressively merged in recursive fashion to create a potentially infinite number of possible structures. This operation underlies what Chomsky also calls *universal grammar* (UG).

Universal grammar is a property of so-called *internal language*, or I-language, which is essentially an internal mode of symbolic thought, and is not itself involved in communication. Chomsky and colleagues have little to say about the origin of I-language, except that it is distinctively and uniquely human, and arose in a single step, even in a single individual (Chomsky 2010)—perhaps the outcome of a mutation. Through a process of externalization, communicative languages (sometimes called external language or E-language) emerge, and may differ widely between different language cultures.

The possibility considered here is that the generative and recursive nature of language, and indeed of thought more generally, derive from spatiotemporal imagination; or as Dor (2015) expresses it, language is “the instruction of imagination.” Generativity in turn is grounded in the hippocampal mechanisms for establishing awareness of location and orientation in space. The generativity of language, then, is not so much a property of language itself as of the underlying thoughts that we use language to convey. Even in the rat, those thoughts may have the beginnings of narrative structure in that hippocampal ripples correspond to sequential trajectories.

The critical difference between this account and that of Chomsky and colleagues, then, is that the internal structure has a long evolutionary history, with basic features evident even in the rat. This approach is clearly more consistent with Darwinian evolution than with the Biblical notion of language as a miracle, a gift of God to Adam. In terms of expressive language itself, then, the question is not how language achieved its fundamental structure, but rather how input–output systems were adapted to enable internal thoughts to be shared between individuals. The answer may lie in the progressive elaboration and refinement of gestural and call systems to map onto those internal thoughts. Some language-like components are evident in the spontaneous gestures of chimpanzees in the wild (e.g., Hobaiter and Byrne 2011), but it is likely that language itself, whether gestural or vocal, emerged gradually through the six-million years separating us from our common ancestry with the chimpanzee, and not within the mere 200,000 years of the existence of *Homo sapiens* (Corballis 2016).

The idea that language originated from gesture is also consistent with a growing view that language, and perhaps thought itself, is a product of spatiotemporal imagination rather than the manipulation of abstract elements.
